# Third harmonic imaging contrast from tubular structures in the presence of index discontinuity

**DOI:** 10.1038/s41598-023-34528-7

**Published:** 2023-05-15

**Authors:** Joséphine Morizet, Nicolas Olivier, Pierre Mahou, Arthur Boutillon, Chiara Stringari, Emmanuel Beaurepaire

**Affiliations:** 1 Laboratory for Optics and Biosciences (LOB), CNRS, INSERM, École polytechnique, Institut Polytechnique de Paris, 91120 Palaiseau, France; 2 SUPA, School of Physics and Astronomy, University of St Andrews, North Haugh, St Andrews, Fife, KY16 9SS UK; 3 Cluster of Excellence Physics of Life, TU Dresden, Dresden, 01062 Germany

**Keywords:** Microscopy, Nonlinear optics, Multiphoton microscopy

## Abstract

Accurate interpretation of third harmonic generation (THG) microscopy images in terms of sample optical properties and microstructure is generally hampered by the presence of excitation field distortions resulting from sample heterogeneity. Numerical methods that account for these artifacts need to be established. In this work, we experimentally and numerically analyze the THG contrast obtained from stretched hollow glass pipettes embedded in different liquids. We also characterize the nonlinear optical properties of 2,2$'$-thiodiethanol (TDE), a water-soluble index-matching medium. We find that index discontinuity not only changes the level and modulation amplitude of polarization-resolved THG signals, but can even change the polarization direction producing maximum THG near interfaces. We then show that a finite-difference time-domain (FDTD) modeling strategy can accurately account for contrast observed in optically heterogeneous samples, whereas reference Fourier-based numerical approaches are accurate only in the absence of index mismatch. This work opens perspectives for interpreting THG microscopy images of tubular objects and other geometries.

## Introduction

Third harmonic generation (THG) microscopy^1–3^ is a multiphoton imaging modality increasingly used for the label-free characterization of interfaces in cells and tissues in combination with fluorescence, with applications ranging from cell and developmental biology to neuroscience^4–12^. THG imaging involves optical frequency tripling near the microscope focus. Under high numerical aperture (NA) focusing conditions, it has been established that THG efficiency is primarily determined by the axial Gouy phase shift of the excitation beam: when the excitation beam is focused in a homogeneous, isotropic, normally dispersive medium, the Gouy phase shift prevents phase matching between the fundamental and harmonic fields over the focal region, resulting in no detected THG  ^1,13–15^. As a result, THG images typically reveal interfaces or optical heterogeneities with sizes of a few hundred nanometers in size, which are highlighted against a dark background. In particular, lipid-water interfaces ^12,16,17^ and myelinated axons ^9,18^ have been identified as efficient sources of contrast. Although this ability to characterize morphology and lipid structures down to the submicron scale in tissues is finding an increasing number of applications, quantitative interpretation of THG data remains challenging due to the non-trivial contrast mechanism involved. It has been shown that the visibility of specific structures depends not only on their optical properties, but also on the interplay between the sample microstructure (size, shape, orientation) and the excitation field distribution (numerical aperture, Gaussian or higher order mode, aberrations) ^15,19–21^. Image interpretation in terms of optical properties and sample geometry therefore relies on numerical simulations that account for interference effects. Complete frameworks have been established for this purpose, using an angular spectrum representation (ASR) of the excitation beam, tensorial nonlinear optics near focus, and propagation of the induced polarization to the detector plane using Green’s Functions ^15,21,22^. However, it has also been found that excitation field distortions caused by micron-scale refractive index heterogeneity have important consequences for CARS ^23^ and THG image contrast ^24^. These effects are expected to occur in most biological samples and are typically not accounted for in ASR/Green models. Recently, it has beeen shown that they can be better described with numerical methods such as finite-difference time-domain (FDTD) modeling of fundamental and harmonic propagation in the near-focus region. We have previously demonstrated the validity of this approach for THG by analyzing the polarized THG (PTHG) contrast near a simple vertical index-mismatched interface ^24^. As a further step towards understanding the THG contrast observed on optically complex structures such as myelinated axons ^9,18,25^ or biological cylindrical structures ^26^, in the present work we analyze the model geometry of an in-plane tubular object made of isotropic material. Following ^27,28^, we consider the case of a stretched hollow glass pipette in the presence of internal and external index liquids. We use experiments and numerical simulations to investigate how the THG and PTHG responses are governed by the combined effect of index mismatch and cylindrical geometry. Experimentally, we stretch borosilicate glass capillaries to a diameter about twenty microns, and use 2,2$'$-thiodiethanol (TDE) as a water-soluble index-matching medium. TDE has been used in fluorescence microscopy studies ^29^ as a tunable-index medium to reduce spherical aberrations due to its high index (1.52) and solubility in water. We estimate the previously unknown third-order nonlinear susceptibility $\chi ^{(3)}$ of TDE by performing z-scan experiments, and we use this value to implement simulations based on the FDTD ^24^ and ASR/Green ^15^ formalisms. We find that the index mismatch has a strong influence on the PTHG contrast of vertical interfaces and on the relative THG visibility of horizontal and vertical interfaces. We show that a FDTD modeling approach can be used to accurately describe THG from cylindrical structures in the presence of index mismatch. This analysis also allows us to resolve some ambiguities and discrepancies between previous studies analyzing THG from cylinders and vertical interfaces ^15,20,24,27^.

## Material and methods

### Pipettes preparation

We used borosilicate capillaries (Phymep, model GC100-10, $\varnothing _{ext}$ = 1.0 mm, $\varnothing _{int}$ = 0.58 mm) which are classically used to perform microinjections in biological samples such as zebrafish embryos. We pulled the capillaries with a micropipette puller equipped with a platinum heating coil (P-1000, Sutter Instrument). By combining high heating temperatures and low stretching forces ^30^, we obtained thin elongated pipette tips with an inner diameter of approximately $12~\mu m$. After removal of the obstructed section at the very end of the pipettes, we filled them with an index liquid ($n=1.33$ or 1.47) containing a fluorescent marker (Rhodamine B, Sigma). The tips of the stretched pipettes were then sealed by melting the glass with a heated resistance. Finally, we placed the pipettes horizontally in Petri dishes with bottom glass plates and immersed them in non-fluorescent index liquids ($n=1.33$ or 1.47). The index liquids used in this study were distilled water ($n=1.33$) and TDE diluted in water ($n=1.47$). The refractive indices of these solutions were controlled with a refractometer at $\lambda =589$ nm. The fluorescent marker inside the pipettes was used to check for proper sealing of the pipette tips after immersion.

### Pipettes imaging and data processing

Multiphoton imaging was performed on a custom-built upright microscope as described in^24^. Excitation was provided by a femtosecond laser source (80 MHz, 1100 nm, 100 fs, Insight X3, SpectraPhysics, USA) and a water immersion objective (25$\times$, 1.05NA, Olympus, Japan). We imaged the pipette tips sequentially with THG and 2PEF contrasts. THG detection was performed in transmission with a high NA condenser and a UV filter (Semrock FF01-377/50). 2PEF was detected in the epi direction with a red filter (Semrock FF01-590/20). The signal level was kept in a range that avoided saturation of the photon-counting detection chain, *i.e.*, less than one photon detected every four laser pulses. We performed two types of acquisitions for each combination of internal and external liquids. First, we acquired *z*-series of *xy* images, where *z* is the optical axis and the incident polarization is parallel to the pipette axis *y*. The *xyz* series were resliced to obtain cross-sectional (*xz*) profiles, and averaged along *y*. In addition, we acquired polarization-resolved *xy* series in the equatorial plane of the pipettes. Lateral and axial sampling were typically $0.23 \mu m$ and $1.0 \mu m$. Polarimetric images were used to extract PTHG profiles by averaging 5 consecutive cross-sectional profiles along the pipette axis. The PTHG modulation was extracted from the polarization series using a unidirectional FFT analysis ^31^.

### $\chi ^{(3)}$ measurements

We estimated the third-order nonlinear susceptibility $\chi ^{(3)}$ of TDE using the classical *z*-scan method ^32–34^. In this approach, horizontal interfaces between materials of known (water/glass) and unknown (e.g. TDE/glass) nonlinear susceptibilities are successively z-scanned across the focus, and the measured amplitudes of the THG maxima are used to infer the $\chi ^{(3)}$ properties of the unknown medium. Samples were prepared by sandwiching a drop of liquid (water, TDE, or diluted TDE) between a microscope slide and a borosilicate glass coverslip (N-BK7, Schott). We then recorded THG profiles by scanning the samples along the *z*-direction from top to bottom with a step of $\Delta z=0.5\,\mu m$. The focal volume successively scanned immersion water, the glass coverslip, and the liquid sample. THG *z*-profiles therefore displayed two peaks: a first peak with a maximum intensity $I_{water/glass}$ was observed at the water/glass interface, and a second peak with a maximum intensity $I_{glass/liquid}$ was observed at the glass/liquid interface. The ratio between these two peak intensities $I_{glass/liquid}\;/I_{water/glass}$ referred as $I^{norm}_{glass/liquid}$ was used to determine $\chi _{liq}^{(3)}$ using Equation 1.

1 $$\begin{aligned} \frac{\chi _{liq}^{(3)}}{\chi _{glass}^{(3)}}=C\left( {1\pm \sqrt{\frac{I^{norm}_{glass/liq}}{I^{norm}_{glass/air}}}}\right) \end{aligned}$$

where $I^{norm}_{glass/air}$ was estimated from a *z*-scan from a sample where air was substituted to an index liquid solution. $\chi _{glass}^{(3)}$ was assessed by applying the previous formula to intensities retrieved from a glass-water *z*-scan experiment and using the value for $\chi _{water}^{(3)}$ established in the literature (See “Results” section).

### Simulations using FDTD model

We performed FDTD simulations^35–37^ of THG from tubular structures immersed in water and TDE by elaborating on the approach described in ^24^. For the simulations, we neglected dispersion by keeping the linear indices constant and we used the $\chi ^{(3)}$ values listed in Table 1, based on reference values for glass and water, and our experimental characterization of TDE properties.

**Table 1:** *n* and $\chi ^{(3)}$ values in the simulations.

Liquid	n	$\chi ^{(3)} (10^{-22}m^{2}V^{-2})$
Water	1.33	1.68
TDE diluted with water	1.47	3.4
Glass (borosilicate)	1.47	1.87

We worked with a commercial FDTD implementation (Lumerical Device suite, Ansys Inc, Canonsburg, PA) to facilitate future comparisons. In this implementation, the electric and magnetic fields are calculated on every point of a 3D grid at successive times by solving discretized Maxwell equations (Eq. 2) for non-magnetic materials ($\overrightarrow{B}=\mu _0\overrightarrow{H}; \overrightarrow{D} = \varepsilon _{0} \varepsilon _{r}\overrightarrow{E}$). The simulations are performed in the time domain, and spectral information can be retrieved using Fourier transforms in a post-processing step.

2 $$\begin{aligned} \overrightarrow{\nabla } .\overrightarrow{D} = \overrightarrow{0} ;\,\, \overrightarrow{\nabla } .\overrightarrow{H} = \overrightarrow{0};\,\, \frac{\partial \overrightarrow{D}}{\partial t} = \overrightarrow{\nabla } \times \overrightarrow{H} \,\, ; \,\, \frac{\partial \overrightarrow{H}}{\partial t} =-\frac{1}{\mu _{0}} \overrightarrow{\nabla } \times \overrightarrow{E} \end{aligned}$$

We used FDTD calculations to estimate THG as a focused beam is laterally scanned in the equatorial plane of cylinder with a 12$\mu$m inner and 20$\mu$m outer diameter. We considered an incoming focused (NA = 1.0) Gaussian beam with a central wavelength of $1.2\;\mu m$ and $50\;nm$ bandwidth, and performed calculations over a focal region spanning $24\times 24\times 16\;\mu m^{3}$ discretized over $20-40\;nm$ steps with a time resolution of $\approx 0.5\;fs$. In the Lumerical software formalism, a nonlinear polarization term is introduced explicitly as follows (Eq. 3):

3 $$\begin{aligned} P_i(t) = \varepsilon _0 \chi ^{(1)}_{ii} E_i(t) + \varepsilon _0 \chi ^{(2)}_{iii} E^2_i(t)+ \varepsilon _0 \chi ^{(3)}_{iiii} E^3_i(t) \,\,\, (i\in \{x,y,z\}) \end{aligned}$$

**Figure 1:**
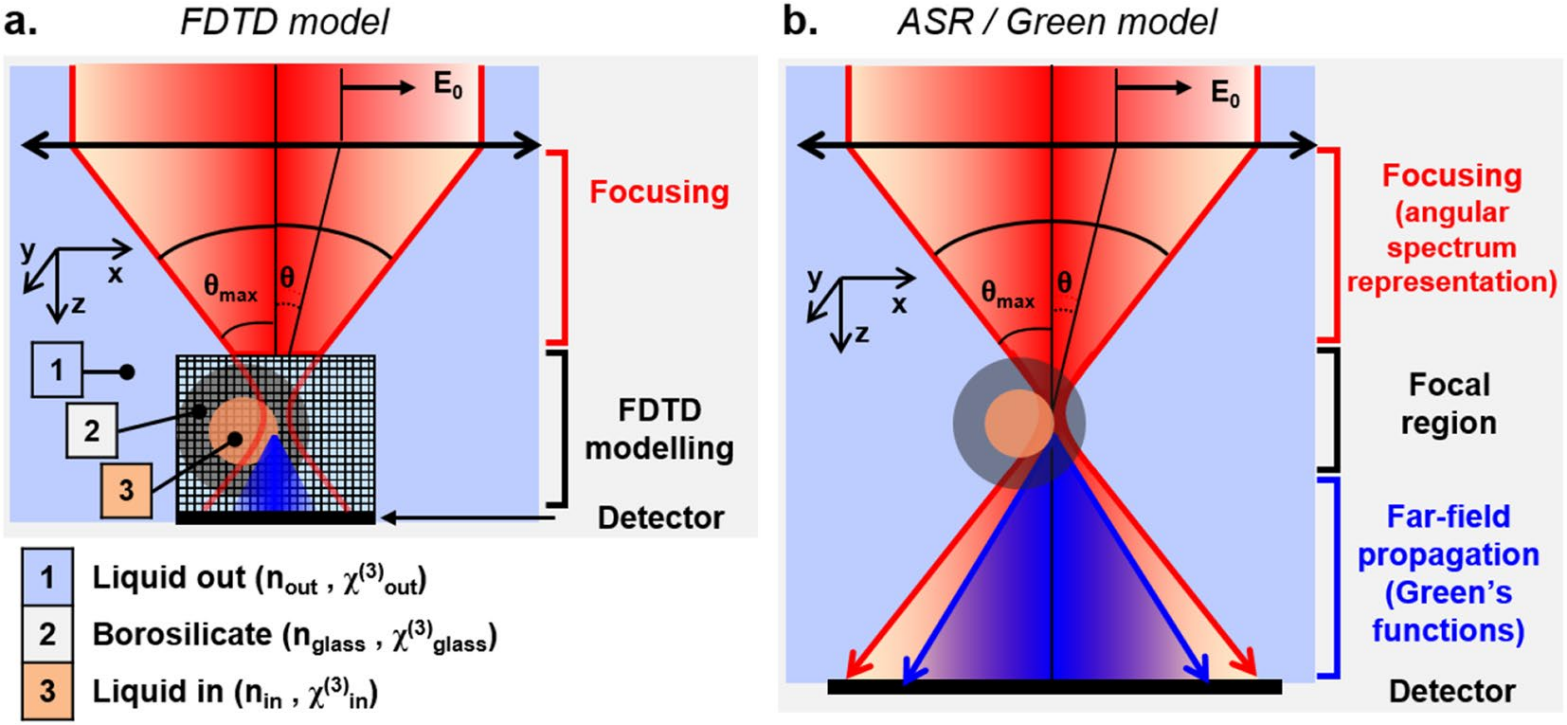
Simulation models. (**a**) Schematic representation of the FDTD approach: the incident field is propagated until the beginning of the FDTD volume assuming no distortion, and this initial distribution as well as the induced response are then numerically propagated through a discretized volume for successive time steps. (**b**) Schematic representation of the ASR/Green three-step strategy: (i) the excitation field if propagated from the objective exit pupil to the focal region, assuming no sample-induced distortions; (ii) the spatially-dependent induced nonlinear polarization is calculated in the focal region; (iii) this polarization is used as a source term and in turn propagated to the detector plane (see ^15,21^).

$\overrightarrow{D}$ is then defined as: ${\overrightarrow{D}(\omega )=\varepsilon _{0} \overrightarrow{E}(\omega )} + \overrightarrow{P}(\omega )$. One limitation of the current version of the software we used is that nonlinear materials can exhibit only diagonal non-zero tensor elements. In order to simulate isotropic materials, we therefore set $\chi _{xxxx}^{(3)}=\chi _{yyyy}^{(3)}=\chi _{zzzz}^{(3)}= \chi _0^{(3)}$ and only considered excitations polarized along $\varvec{x}$ or $\varvec{y}$. Since storing the entire simulated fields would require several tens of gigabytes per simulation, we instead considered a 2D array of detectors in a slab of linear material located at the end of the simulation domain, and computed the integrated intensity around 1/3 of the incoming wavelength ($385-415\;nm$). Figure 1.a summarizes the simulation geometry and parameters. FDTD simulations were performed on a Dell Precision 7920 (2 × Intel 6140 CPUs, 384 GB RAM, DDR4 2666 MHz) running Lumerical version 2019b, 2020a, or 2021R2.4 with a typical computation time of $\approx$ 2 hours per condition.

### Simulations using ASR/green model

For comparison, we performed ASR/Green’s function calculations of THG from the same geometries using the method described in previous studies ^15,20–22,34^. This simulation strategy has been used to simulate nonlinear microscopy in the absence of aberrations. It relies on an angular spectrum representation (ASR) to calculate the excitation field distribution near the focus, assuming the same linear index everywhere ^38,39,^ and on Green’s functions ^39,40^ to propagate the nonlinear response from the focal region to the detector plane (again, assuming constant linear indices). Figure 1b summarizes the simulation geometry and parameters.

## Results

### THG from interfaces as a function of orientation and index mismatch

**Figure 2:**
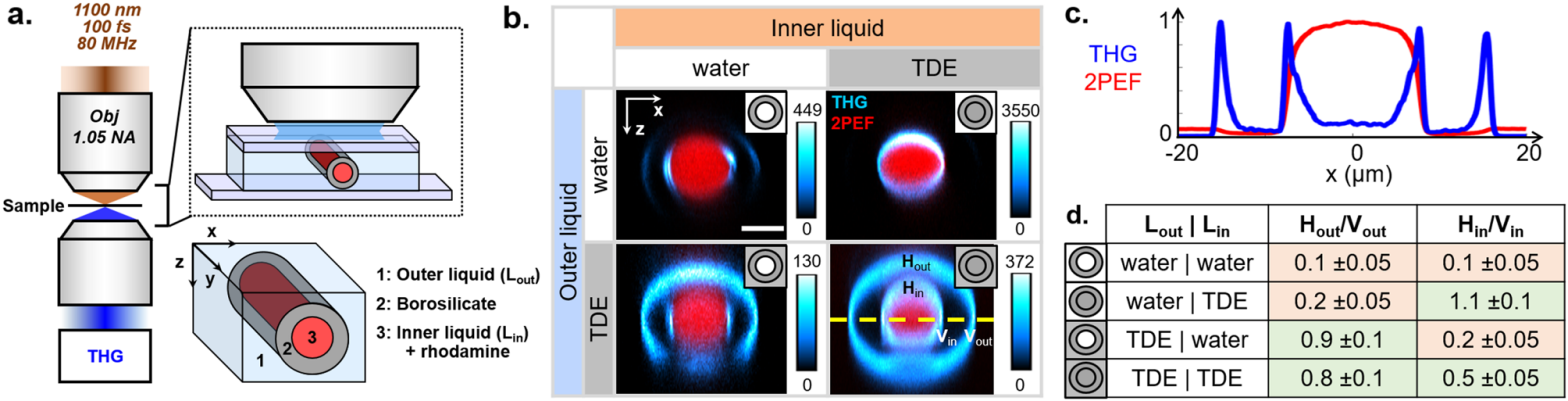
THG contrast from stretched glass pipettes in different immersion media. (**a**) Experimental setup. A stretched glass pipette is filled with a fluorescent inner liquid ($L_{in}$), sealed, and placed in a closed observation chamber filled with a second liquid ($L_{out}$). $L_{in}$ and $L_{out}$ can be either water or a TDE solution index-matched to the pipette. THG signals are detected in transmission. (**b**) XZ reprojections of 3D images taken near the pipettes tips for different combinations of $L_{in}$ and $L_{out}$. Blue: THG signals. Red: fluorescence signals. The plot shows THG and 2PEF intensity profiles in the pipette equatorial plane for the TDE/TDE case. Scale bar: $10 \mu m$. (**c**) Relative visibility of the horizontal (H) and vertical (V) interfaces for all experimental conditions.

We first investigated the THG visibility of internal, external, horizontal and vertical interfaces as a function of index mismatch. We used TDE as a water-soluble index medium and diluted it (26%) to approximately match the refractive index of borosilicate glass (1.47 at 589 nm). We recorded *xz* sections of pipettes in four different conditions where the internal and external liquids were either index-mismatched (water) or index-matched (diluted TDE) with the borosilicate pipettes. Rhodamine was diluted in the internal liquid in order to verify that the pipette tips were efficiently sealed, and to easily identify the internal part of the pipettes. The experimental geometry and typical images are shown in Fig. 2. An obvious phenomenon is that the visibility of the different interfaces depends on both their orientation and the index mismatch. More specifically, we observed that:

- Glass-TDE interfaces efficiently produce THG despite index matching. This suggests that TDE and glass have different nonlinear susceptibility $\chi ^{(3)}$. In such case, standard models of THG microscopy near interfaces ^15^ predict that the observed signal scales as $(\chi _{glass}^{(3)}-\chi _{TDE}^{(3)})^2$.
- Glass-TDE horizontal interfaces are more visible than glass-water horizontal interfaces. This suggests that TDE and water also have different $\chi ^{(3)}$.
- H-interfaces and V-interfaces have similar visibility ($H/V \approx 0.5-1$) in index-matched cases, whereas H-interfaces are less visible than V-interfaces ($H/V \approx 0.1$) in index-mismatched cases. This is a less intuitive observation, which suggests that focus distortion contributes to THG image contrast, as we recently analyzed in the case of index-mismatched vertical interfaces ^24^.
- Vertical glass-water interfaces sometimes exhibit double THG peaks along the direction transverse to the optical axis. This is consistent with our recent work using similar materials ^24^.

### Third-order nonlinear susceptibility $\chi ^{(3)}$ of TDE

**Figure 3:**
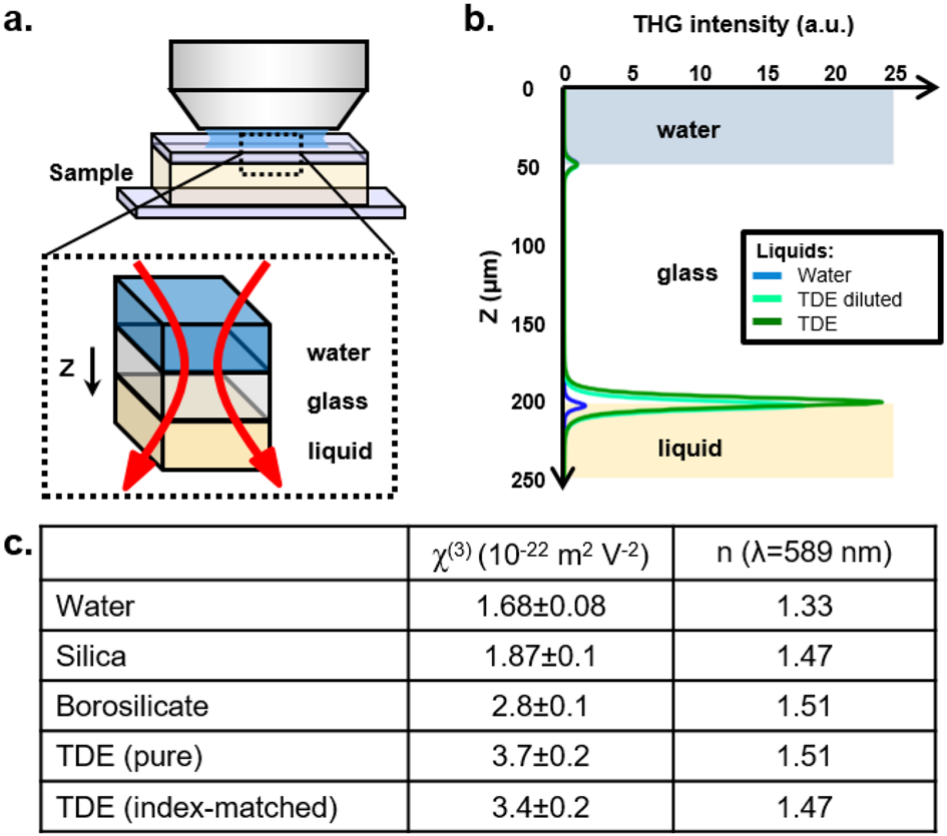
Experimental determination of $\chi ^{(3)}$ for TDE. (**a**) A liquid with known (water) or unknown (TDE) nonlinear properties was placed below a glass coverslip, and THG z-scans were recorded across the first (immersion water / coverslip) and second (glass / liquid) interfaces (see text). (**b**) Recorded z-scans profiles. (**c**) Experimentally determined $\chi ^{(3)}$ values for TDE solutions, and $\chi ^{(3)}$ values for water and glass used in the calculations.

The observation that glass-TDE interfaces produce strong THG despite index matching is intriguing. However, the third-order nonlinear susceptibility $\chi ^{(3)}$ of TDE is not described in the literature. In order to better understand the origin of the glass-TDE signal and more generally to numerically investigate the THG contrast from pipettes, we determined experimentally the $\chi ^{(3)}$ values for pure and diluted TDE using the classical z-scan technique (see Methods). The experimental arrangement and results are shown in Fig. 3. We found that the $\chi ^{(3)}$ of pure TDE at 1100 nm is particularly high, namely on the order of $3.7\pm 0.2 \times 10^{-22}m^{2}V^{-2}$. The $\chi ^{(3)}$ of 26%-diluted TDE at 1100 nm is still $3.4\pm 0.2 \times 10^{-22}m^{2}V^{-2}$, *i.e.* higher than that of silica ($1.9\pm 0.1 \times 10^{-22}m^{2}V^{-2}$), borosilicate ($2.8\pm 0.1 \times 10^{-22}m^{2}V^{-2}$) or lipids ($2.6\pm 0.1 \times 10^{-22}m^{2}V^{-2}$)^17^. This surprisingly high value accounts for the visibility of glass-TDE interfaces, since the THG intensity observed near an index-matched interface scales as the squared difference between the nonlinear susceptibilities of the two media: $THG \propto \Delta (\chi ^{(3)})^2$. These measurements also suggest that TDE could be used as a contrast agent in THG imaging.

**Figure 4:**
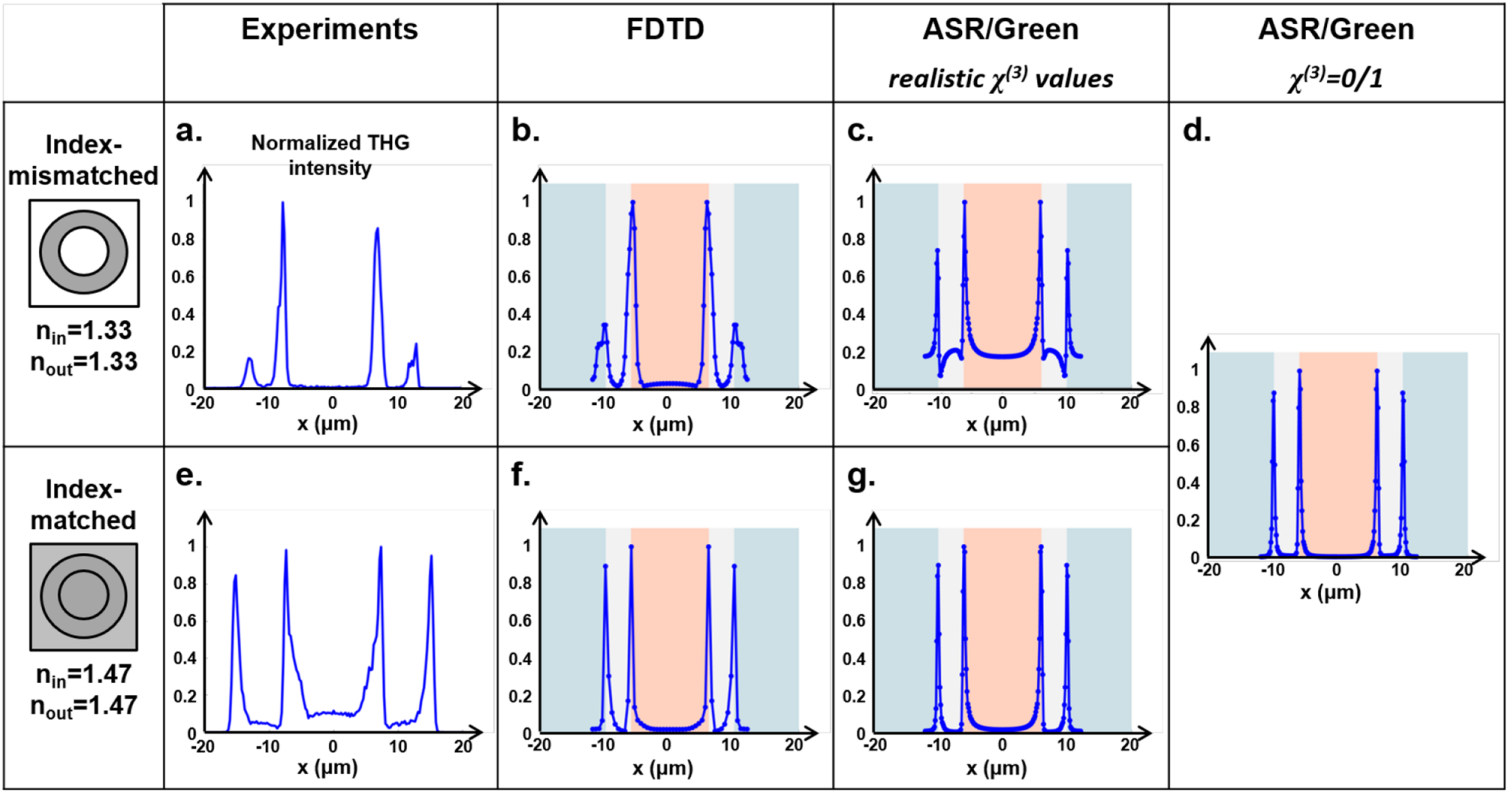
THG contrast from vertical interfaces in the equatorial plane of a horizontal cylinder. Top row, measured (**a**) and simulated (**b**–**d**) THG profiles in the case of a glass pipette immersed in water. Bottom row, measured (**e**) and simulated (**f**, **g**) THG profiles in the case of a glass pipette in TDE. In the index-mismatched case, the relative visibility of the internal and external interfaces is correctly predicted by FDTD simulations (**b**) and not by ASR/Green simulations (**c**, **d**). Both models work well to describe index-matched samples (**f**, **g**).

### Modelling THG contrast in the equatorial plane

Using this measured value of the third-order nonlinear susceptibility of TDE, we implemented FDTD simulations of the THG contrast as the excitation focus is laterally scanned in the equatorial plane of a 20 $\mu m$ diameter glass pipette immersed either in TDE or in water. We also performed corresponding ASR/Green model-based calculations of the same geometry. Figure 4 presents experimental and simulated profiles obtained in the index-mismatched (glass-water) and index-matched (glass-TDE) cases. The index-mismatched case is of particular interest, because focus distortions can produce non-trivial THG contrast, in particular near interfaces ^24^. One first observation is that internal vertical interfaces experimentally produce 3-to-5 times more THG than external interfaces (Fig. 4a). This phenomenon is likely due to the fact that the scanned beam is more aberrated when focused on the outer edge of the pipette in its equatorial plane, resulting in reduced excitation intensity. Interestingly, this THG profile is reproduced by the FDTD-based calculation (Fig. 4b). This confirms that FDTD is a valid formalism for reproducing the propagation of the excitation field in an index-mismatched cylindrical geometry. In contrast, ASR/Green calculations predict that inner and outer THG peaks should have similar amplitudes (Fig. 4c), in contradiction with the experimental observation. We also note that the shape of the peaks predicted by ASR/Green calculations is distorted when using realistic values for $\chi ^{(3)}$. This is a numerical artifact due to the fact that the signal scales as the difference between the squared $\chi ^{(3)}$ ($\Delta \chi ^{(3)}$) while the background signal due to the finite integration volume scales as the average value of the squared $\chi ^{(3)}$, so realistic values add an interference term between these two contributions. This artifact is classically avoided in published studies ^15,20^ by numerically increasing the $\chi ^{(3)}$ contrast and setting $\chi ^{(3)}$ = 0 or 1. As shown in Fig. 4d, this numerical trick removes the artifacts. However, this is at the cost of using unrealistic $\chi ^{(3)}$ values, which limits the quantitative nature of the numerical predictions. In the index-matched case of a pipette immersed and filled with TDE (Fig. 4e–g), inner and outer THG peaks have similar amplitudes, and all simulation strategies produce accurate predictions.

**Figure 5:**
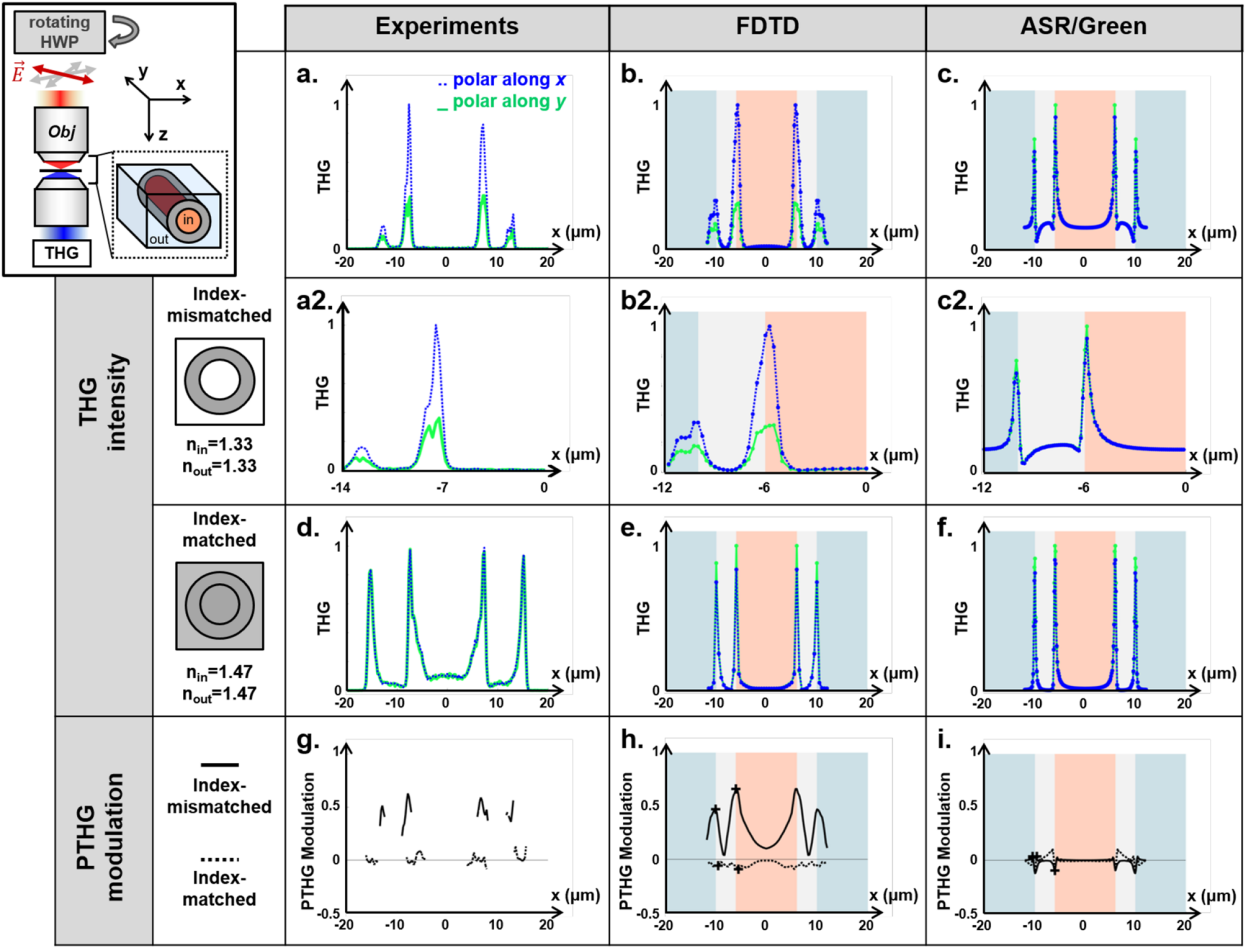
Polarized THG contrast from vertical interfaces at the equator of a cylindrical sample. Top row, measured (**a**) and simulated (**b**, **c**) THG profiles in the case of a glass pipette immersed in water. Second row (**a2**–**c2**), zoomed-in views of the left-side peaks. Third row, measured (**d**) and simulated (**e**, **f**). Bottom row, PTHG modulation amplitudes measured from the experimental data (**g**), FDTD (**h**) and ASR/Green simulations (**i**). Index mismatch results experimentally in a strong PTHG modulation (50–60% with more THG for an incident polarization perpendicular to the interface) and in lateral THG peak doubling near vertical interfaces (**a2**, **g**). These phenomena are predicted by FDTD calculations (**b2**, **h**) and not by the ASR/Green model (**c2**, **i**). Both models perform well in the case of an index-matched samples.

### PTHG contrast in the presence of index discontinuity

We then explored experimentally and numerically the polarization-resolved THG contrast in the equatorial plane of the pipettes in the two previous cases (Fig. 5): we recorded THG profiles with an excitation polarization in the directions perpendicular (*x* direction) and orthogonal (*y* direction) to the pipette axis, in index-mismatched (water) and index-matched (TDE) environments. The experimental observations are somehow intriguing: in line with previous reports^24^, index-mismatched vertical interfaces produced a strong PTHG modulation ($>50\%$), with maximum THG observed when the polarization is orthogonal to the interface (Fig. 5a,a2); in contrast, we observed a greatly reduced PTHG modulation ($<8\%$) near index-matched interfaces (Fig. 5d,g). This phenomenon is necessarily caused by polarization-dependent aberrations, and should of course be taken into account in the context of PTHG microscopy. Fortunately, this modulation dependence on index mismatch is reproduced by the FDTD calculations (Fig. 5b,b2,e,h). In contrast, the phenomenon is not reproduced by ASR/Green calculations (Fig. 5c,c2,f,i).

**Figure 6:**
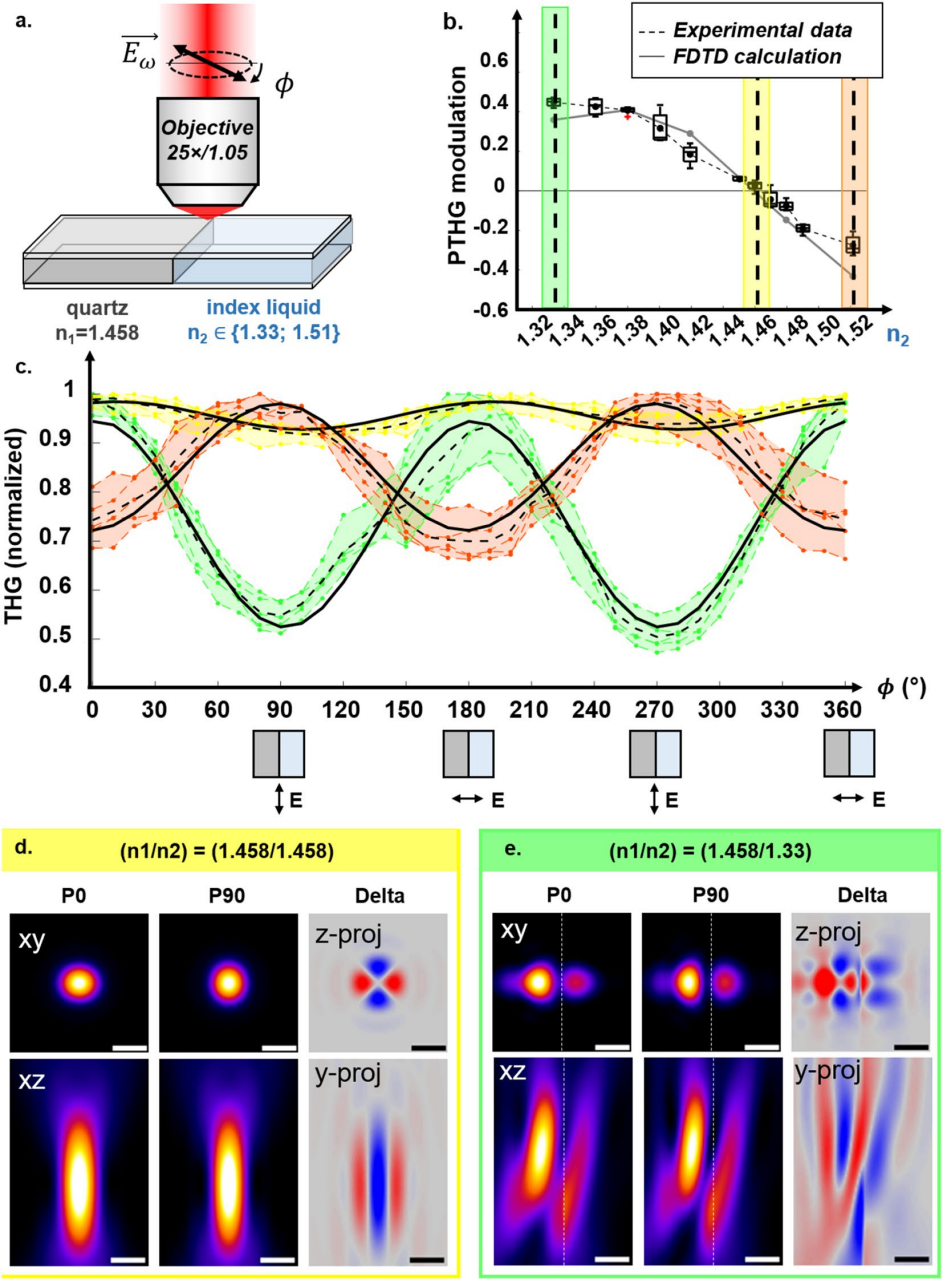
Index mismatch can invert PTHG modulation on a vertical interface. (**a**) Experimental geometry considered: P-THG signals were measured on a vertical interface between a quartz coverglass and a mixture of water+TDE (**b**) PTHG modulation (red: experiment, blue: FDTD) as a function of the index of refraction of the water-TDE mixture. (**c**) experimental measurements showing THG signals as a function of the incident polarization for the 3 different conditions (water, index-matched and pure TDE) identified in (**b**). (**d**) and (**e**): FDTD calculations of the intensity distribution near a *yz* vertical interface for an incident polarization parallel (P0) or perpendicular (P90) to the interface, for index-matched and index-mismatched conditions. The third column displays the P90-P0 difference averaged along *z* and *y* (LUT: red, P0>P90; blue, P90>P0). Scalebars= 1$\mu$m.

### PTHG modulation inversion on vertical interfaces

Looking more carefully at the index-matched case, we note that both simulation models predict a limited dependence of THG on polarization (modulation amplitude $\le 15$%) with maximum signal obtained for a polarization *parallel* to the interface ^15^. In the following, we will speak of positive (resp. negative) PTHG modulation in cases where maximum THG is obtained for an incident polarization orthogonal (resp. parallel) to the interface. In experiments on index-matched pipettes, we did experimentally confirm a greatly reduced PTHG modulation near vertical interfaces, but we did not unambiguously measure the small negative modulation predicted by the simulations. This discrepancy may be due to non-perfect index matching, since we measured the optical indices at a different wavelength than the one use for imaging. Since PTHG modulation near a vertical interface appears to strongly depend on the optical contrast between the two materials, we investigated this effect more precisely. We revisited experimentally and numerically the case of a simple vertical interface^24^, this time with various values of the index mismatch. We measured PTHG modulation at the interface between glass and TDE solutions of increasing concentrations, with indices ranging from 1.33 (pure water) to 1.52 (pure TDE). For increasing concentrations of TDE, we measured (Fig. 6) PTHG modulation values ranging from largely positive (+45%) to null and even to increasingly negative (-25%) values. This previously undescribed phenomenon is well reproduced by the FDTD simulations for all experimental cases (Fig. 6). In the well-studied case of perfect index matching, a small PTHG negative modulation of approximately 10-15% is predicted by the classical ASR model ^15^, which is due to the presence of the interface that breaks the circular symmetry of the problem. In the case of an index-mismatched interface, the physical interpretation is more complicated, as additional mechanisms alter THG efficiency. Most importantly, propagation along an index-mismatched vertical interface can severely distort the excitation field distribution at the sub-wavelength scale, and such aberrations are polarization-dependent. As an illustration, Fig. 6 shows polarization-resolved views of the distorted excitation field distribution in the *xy* and *xz* planes, for both matched ($n_1=n_2=1.458$) and mismatched ($n_1=1.458,\, n_2=1.33$) cases. Aberrations introduce a polarization-dependent field asymmetry near the focal region, and therefore a polarization-dependent source of THG signal. THG efficiency is then governed by the interplay between this altered field distribution and the spatial distribution of nonlinear susceptibility $\chi ^{(3)}$ of the sample. In the most general case, PTHG modulation near a vertical interface is possibly a function of linear index mismatch, $\chi ^{(3)}$ distribution, as well as materials anisotropy ^41^ near focus. As indicated by our measurements and simulations, this complexity can in principle be investigated with advanced numerical methods. Finally, double lateral THG peaks were occasionally observed near index-mismatched vertical interfaces (Figs. 2b, 5a2), and were properly predicted by FDTD simulations (Fig. 5b2), as discussed in a previous study ^24^.

### Relative visibility of horizontal and vertical interfaces

**Figure 7:**
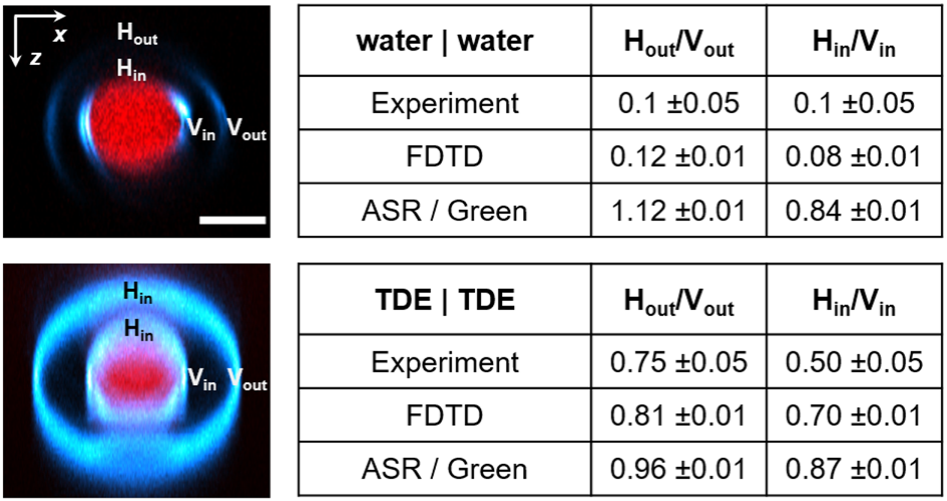
THG contrast from horizontal and vertical interfaces. Relative visibility of horizontal (H) and vertical (V) interfaces in the the index-mismatched (water, top) and index-matched (TDE, bottom) configurations. Left, XZ projections of experimental data. Right, summary of experimental and calculated H/V ratios for internal and external interfaces. Index mismatch increases the relative visibility of vertical interfaces, as predicted by FDTD calculations.

Finally, we analyzed the relative visibility of horizontal (H) and vertical (V) interfaces, summarized in Fig. 7. Experiments (Fig. 2c) showed that the visibility ratio between H-interfaces and V-interfaces is low ($H/V = 0.1 \pm 0.01$) in index-mismatched cases (glass/water interfaces), and gets close to 1 ($H/V \approx 0.5-1$) in index-matched cases (glass/TDE). We ran FDTD and ASR/Green calculations of THG by horizontal and vertical interfaces in a tubular geometry Fig. 7, averaged for $x-$ and $y-$ polarizations. We found that FDTD calculations properly reproduced the index-mismatch (glass/water) experiment ($H/V = 0.1 \pm 0.02$), in contrast with the ASR/Green simulations ($H/V \approx 1 \pm 0.2$). In the index-matched case (pipettes immersed in TDE), both models produced reasonable predictions ($H/V \approx 0.7-9$). The higher relative visibility of vertical index-mismatched interfaces could be partly due to the fact that this geometry results in a severe distortion of the focused field distribution (see e.g. Fig. 6e, in turn creating an effective symmetry breaking resulting in more efficient THG (i.e. less efficient Gouy shift-induced destructive interference). In a sense, THG microscopy may be viewed as a aberration detector in which signal can be enhanced by focus distortions, at least up to a certain amount of aberration. Our data indicate that this phenomenon should be tractable with FDTD calculations.

## Discussion

In this work, we have shown that an FDTD modeling strategy can accurately account for the THG contrast observed in optically heterogeneous structures, while the faster classical Green function-based simulation methods are reliable only in the absence of index mismatch. This analysis opens perspectives for the analysis of the interplay between sample geometry ^42^, excitation NA ^19^, beam profile ^20,21^, and field distortion induced by index heterogeneity ^23,24^ in THG microscopy - and ultimately to develop more quantitative interpretations of THG images, with the possibility of extracting submicron structural parameters. Our data show that the polarized THG contrast near interfaces is particularly sensitive to index mismatch. An interesting observation is that index-mismatched vertical glass-water interfaces, often encountered in microscopy experiments, produce a strong PTHG modulation ($>50$%) with maximum THG observed when the polarization is orthogonal to the interface, whereas index-matched interfaces produce a weak inverted modulation with maximum THG when the polarization is parallel to the interface, as originally predicted by Green-based simulations ^15^. This phenomenon is caused by polarization-dependent field-distributions, and can lead to misinterpretations if the images are not analyzed with an appropriate model. This is a particularly important observation in the context of PTHG microscopy. Indeed, it should be emphasized that these strong PTHG modulations are demonstrated here in the case of *isotropic* materials, and they can be stronger than the contribution due to materials anisotropy (linear and nonlinear). The interpretation of some PTHG^41,43^ and possibly PCARS^44–46^ experiments in optically heterogeneous samples should therefore probably be reconsidered in light of these findings. Fortunately, our data also show that these subtle effects can be analyzed with numerical FDTD approaches, even in the case of a complex sample geometry such as a double cylinder. These result pave the way for accurate modeling of PTHG from arbitrary objects. In particular, the numerical approach presented here could be extended to account for optical anisotropy, which would be appropriate for the analysis of THG from organized materials such as ordered lipid assemblies^41,47^, corneal stroma ^48,49^, myelin ^9,18,25^, or biocrystals ^43,50^.

## Data Availability

The simulation code that support the findings of this study are openly available on GitHub at: https://github.com/LaboratoryOpticsBiosciences/THG_PIPETTES.
